# Screening the methanol extracts of some Iranian plants for acetylcholinesterase inhibitory activity

**Published:** 2009

**Authors:** A. Gholamhoseinian, M.N. Moradi, F. Sharifi-far

**Affiliations:** 1 *Department of Biochemistry, Medical School and Kerman Physiology Research Center, Kerman University of Medical Sciences, Kerman, I.R.Iran*; 2 Department of Pharmacognosy, School of Pharmacy, Kerman university of Medical Sciences, Kerman, I.R.Iran

**Keywords:** Acetylcholinesterase, Inhibitor, Levisticum officinale, Bergeris integrima, Rheum ribes

## Abstract

Acetylcholinesterase (AChE) is the main enzyme for the breakdown of acetylcholine. Nowadays, usage of the inhibitors of this enzyme is one of the most important types of treatment of mild to moderate neurodegenerative diseases such as Alzheimer’s disease. Herbal medicines can be a new source of inhibitors of this enzyme. In this study we examined around 100 different plants to evaluate their inhibitory properties for AChE enzyme. Plants were scientifically identified and their extracts were prepared by methanol percolation. Acetylcholinesterase activity was measured using a colorimetric method in the presence or absence of the extracts. Eserine was used as a positive control. Methanol extracts of the *Levisticum officinale, Bergeris integrima* and *Rheum ribes* showed more than 50% AChE inhibitory activity. The inhibition kinetics were studied in the presence of the most effective extracts. *L. officinale* and *B. integrima* inhibited AChE activity in a non-competitive manner, while *R. ribes* competitively inhibitied the enzyme as revealed by double-reciprocal Linweaver-Burk plot analysis. Under controlled condition, K_m_ and V_max_ values of the enzyme were found to be 9.4 mM and 0.238 mM/min, respectively. However, in the presence of *L. officinale, B. integrima*, and *R. ribes* extracts, V_max_ values were 0.192, 0.074 and 0.238 mM/min, respectively. Due to the competitive inhibition of the enzyme by *R. ribes* extract, the K_m_ value of 21.2 mM was obtained. The concentration required for 50% enzyme inhibition (IC50 value) was 0.5, 0.9, and 0.95 mg/ml for the *L. officinale, B. integrima* and *R. ribes* extracts, respectively. The IC50 of the eserine was determined to be 0.8 mg/ml.

## INTRODUCTION

The main role of acethylcholinestrase (AChE) is to rapidly hydrolyze acetylcholine at the cholinergic synapses, ending the transmission of nerve impulses (1). The use of AChE inhibitors in order to enhance cholinergic function in the brain is the main strategy in treatment of Alzheimer’s disease (AD) which is characterized by loss or decline in memory and cognitive impairment (2,3). AD is the most common cause of dementia in the elderly and is responsible for 60 to 80 percent of the cases (2). Degeneration and loss of basal forebrain cholinergic innervation is accepted as a major cause of cognitive impairment and memory loss for the disease (4–6).

The pathological hallmarks of AD are the senile plaques and neurofibrillary tangles. The accumulation of plaques and tangles, and the progression of other pathological processes, leads to a massive neuronal loss, which is usually preceded by synapse loss (1). Several AChE inhibitors such as tacrine, donepzil, rivastigmine and galanthamine, are available for the treatment of mild to moderate AD (7). Although the use of these drugs are beneficial in the treatment of AD symptoms, they may also cause some adverse side effects (8). The most common side effects of these drugs include: anorexia, diarrhea, fatigue, nausea, muscle cramps as well as gastrointestinal, cardiorespiratory, genitourinary and sleep disturbances (6,8). Therefore, cheaper and safer AChE inhibitors with higher efficacy, bioavailability and fewer side effects, particularly from natural sources, have been extensively investigated and research should be continued.

## MATERIALS AND METHODS

### 

#### Plant material

Different parts of all plants such as flowers, fruits, seeds, aerial parts and roots were collected from various provinces of Iran or obtained from the medical herbal markets in Kerman city. Scientific names of the collected plants were authenticated. A voucher specimen of each plant was retained in the herbarium in the Herbal Medicine Research Center, Faculty of the Pharmacy, Kerman University of Medical Sciences, Kerman, Iran.

#### Extraction of plant material

Each plant was air dried in the dark, and grounded into fine powder. The powdered materials (20 g) were extracted with 200 ml of absolute methanol for 24 h at room temperature. The suspensions were then filtered and air-dried. The extracts were stored at -20 °C until being used (9).

#### Chemicals

Acetylthiocholine iodide (ATCI), Electric eel acetylcholinesterase and 5-5’-thiobis-2-nitrobenzoic acid (DTNB) were purchased from Sigma (USA). Eserine was obtained from Merck (Germany). All other reagents were of analytical grade.

Acetylcholinesterase activity assay

The AChE activity was measured according to the method developed by Ellman et al. (10). This method employs ATCI as a synthetic substrate for AChE. In this procedure 10 μl of methanol extract containing 50 μg of crude extract was added to the reaction mixture containing 20 μl of enzyme solution (0.1 U/ml) and 950 μl sodium phosphate buffer (pH 8, 0.1 M). Reaction mixture was incubated for 15 min at 25 °C. Then, 10 μl of DTNB (10 mM) was added and the reaction initiated by the addition of substrate (10 μl of ATCI 14 mM solution). Based on this procedure, ATCI is broken down to thiocholine and acetate by the enzyme and thiocholine is reacted with DTNB to produce yellow color. The intensity of yellow color was measured at 410 nm after 10 min. Eserine (20 μl of 0.1 mM solution in phosphate buffer) was used as positive control. The percentage of enzyme inhibition was calculated using the following formula(11).

$$\mathrm{Inhibition}\%\;=\;100-\left[A_{t}/A_{c}\;\times \;100\right]$$

where, A_t_ is the absorbance of the tested extract and A_c_ is the absorbance of the standard control.

#### Kinetic study

In order to elucidate the type of inhibition of the effective extracts, the enzyme activity was measured in the presence of an increasing concentrations of ACTI (2-20 mM), and in the presence or absence of two concentrations of each extract (4 and 8 mg/ml). Inhibition mode was determined by double-reciprocal Lineweaver-Burk plot analysis of the data resulted from enzyme assays containing various concentrations of ACTI and the extracts.

## RESULTS

### Plants with acetylcholinesterase inhibitory effect

Among all plants examined, *B. integrima, L. officinale* and *R. ribes* showed the strongest inhibition on the enzyme activity (80.2, 97.6 and 72.4%, respectively). *Alhagi camelorum, Marrubium anisodon, Vaccinium arcto-staphilus, Peganum harmala, Rosa damascene and Valeriana hispida, Myrtus communis, Nepta saccharata and Quercus infectoria* showed inhibitory effect between 20-50%. The other plants showed inhibitory effect less than 20% or had no effect on enzyme activity. Acetylcholinesterase inhibitory activity of all plants is shown in Table 1.

**Table 1:** Acetylcholinesterase inhibitory activity of plants.

Plants name	Family	Used part	Inhibition %
*Achillea eriophora*	Asteraceae	Aerial parts	N.E
*Acantholepis orientalis*	Asteraceae	Aerial parts	N.E
*Achillea phillea*	Composiatae	Aerial parts	9
*Achillea wilhelmsii*	Asteraceae	Aerial parts	0.1
*Acroptilon repens*	Asteraceae	Aerial parts	N.E
*Alhagi camelorum*	Fabaceae	Aerial parts	29.7
*Anacardium occidentale*	Anacardiaceae	Rhizomes	4.6
*Alpinia officinarum*	Zingiberaceae	Rhizomes	0.4
*Althaea officinalis*	Malvaceae	Flowers	1.7
*Apium graveolens*	Umbelliferae	Leaves	4.7
*Arctium lappa*	Asteraceae	Roots	N.E
*Artemisia santolina*	Asteraceae	Aerial parts	4.9
*Biebersteinia multifida*	Berberdaceae	Aerial parts% fruits	2
*Berberis integrima*	Berberdaceae	Roots	80.2
*Bunium persicum*	Apiaceae	Seeds	16.8
*Camellia sinensis*	Theaceae	Leaves	N.E
*Cannabis sativa*	Cannabaceae	Seeds	N.E
*Cardaria draba*	Brassicaceae	Aerial parts% flowers	N.E
*Carthamus oxyacantha*	Asteraceae	aerial parts	N.E
*Chaerophyllum khorassanicum*	Apiaceae	Aerial parts	N.E
*Cichorium intybus*	Asteraceae	Roots	12.7
*Cinnamomum zeylanicum*	Lauraceae	Derm	0.5
*Citrus aurantium*	Rutaceae	Flowers	7.4
*Citrus sinensis*	Rutaceae	Fruits hull	1.2
*Clematis officinalis*	Ranunculaceae	Aerial parts	18
*Convolvulus pilosellaefolius*	Concolvulaceae	Aerial parts	10.4
*Cordia mixa*	Boraginaceae	Fruits	9
*Crocus sativa*	Iridaceae	Leaves	N.E
*Cuminum cyminum*	Apiaceae	Seeds	9.9
*Eremostachys laciniata*	Lmiaceae	Whole the plant	2.2
*Eremurus persicus*	Liliaceae	Aerial parts	7
*Eremurus persicus*	Liliaceae	Flowers	N.E
*Eremurus persicus*	Liliaceae	Fruits	N.E
*Eucaliptus galbie*	Myrtaceae	Leaves	N.E
*Euphorbia hebecarpa*	Euphorbiaceae	Aerial parts% flowers	N.E
*Falcaria vulgaris*	Umbelliferaceae	Aerial parts	6.6
*Ferula assafoetida*	Apiaceae	Aerial parts% flowers	N.E
*Ferula oopoda*	Apiaceae	Aerial parts	N.E
*Ferulago angulata*	Apiaceae	Aerial parts	5.3
*Ficus carica*	Moraceae	Leaves	3.7
*Foeniculum vulgare*	Apiaceae	Fruits	N.E
*Francoeuria undulata*	Asteraceae	Aerial parts	3.1
*Fumaria parviflora*	Fumariaceae	Aerial parts	15.5
*Glycyrrhiza glabra*	Fabaceae	Aerial parts	N.E
*Gundelia tournefortii*	Asteraceae	Aerial parts	N.E
*Heracleum persicum*	Apiaceae	Fruits	6.5
*Hibiscus gossypifolius*	Malvaceae	Flowers	0.5
*Hyoscyamus senecionis*	Solanaceae	Aerial parts% flowers	3.5
*Laurus nobilis*	Lauraceae	Leaves	6.5
*Lawsonia inermis*	Lythraceae	Leaves	8.6
*Levisticum officinale*	Apiaceae	Roots	97.6
*Linum usitatissimum*	Liliaceae	Seeds	N.E
*Malva sylvestris*	Malvaceae	Flowers	1.5
*Marrubium anisodon*	Lamiaceae	Aerial parts	27.7
*Matricaria aurea*	Asteraceae	Flowers	N.E
*Mentha longifolia*	Lamiaceae	Aerial parts	N.E
*Mentha piperita*	Lamiaceae	Leaves	4.2
*Myrtus communis*	Myrtaceae	Leaves	20.4
*Nepeta crispa*	Lamiaceae	Aerial parts	6
*Nepeta saccharata*	Lamiaceae	Whole the plant	21.5
*Nigella sativa*	Ranunculaceae	Seeds	N.E
*Origanum majorana*	Lamiaceae	Whole the plant	7.9
*Otostegia persica*	Lamiaceae	Aerial parts	0.06
*Outreya carduiformis*	Asteraceae	Aerial parts	12.3
*Peganum harmala*	Nitrariaceae	Aerial parts	29.8
*Piper nigrum*	Pipereaceae	Fruit	3.7
*Pistacia vera*	Anacardiaceae	Fruits hull	5.5
*Punica granatum*	Lythraceae	Fruits hull	11.5
*Quercus infectoria*	Fagaceae	Galls	21.4
*Rheum ribes*	Polygonaceae	Rhizomes	72.4
*Rosa damascene*	Rosaceae	Floret	27.9
*Rosmarinus officinalis*	Lamiaceae	Aerial parts	N.E
*Rubia tinctorium*	Rubiaceae	Roots	8.8
*Salix alba*	Salicaceae	Aerial parts	3.5
*Salvadora persica*	Salvadoraceae	Wood	19
*Salvia rhytidea*	Lamiaceae	Whole the plant	N.E
*Sanguisorba minor*	Rosaceae	Aerial parts	18
*Scorphularia frigid*	Scorophulariaceae	Aerial parts	2.9
*Sizigium aromaticus*	Caryophyllaceae	Floret	N.E
*Solanum dulcamara*	Solanaceae	Fruits	4.8
*Sonchus asper*	Asteraceae	Aerial parts	N.E
*Sophora alopecuroides*	Fabaceae	Aerial parts	3
*Stachys inflate*	Lmiaceae	Aerial parts	5.2
*Stachys lavandulifolia*	Lamiaceae	Aerial parts	7.4
*Terminalia chebulla*	Combretaceae	Fruits	N.E
*Teucrium polium*	Lamiaceae	Aerial parts	10
*Teucrium scordium*	Lamiaceae	Aerial parts	N.E
*Thymus serpyllum*	Lamiaceae	Aerial parts	N.E
*Tragopogon carcifolius*	Compositae	Areial parts	4.7
*Trigonella foenum graecum*	Fabaceae	Seeds	1.8
*Urtica dioica*	Urticacea	Aerial parts	2.9
*Verbascum kermanensis*	Scrophulariaceae	Leaves	2.7
*Verbascum songaricum*	Scrophulariaceae	Aerial parts	N.E
*Zataria multiflora*	Lamiaceae	Aerial parts	8.2
*Zhumeria majdae*	Lamiaceae	Leaves	8.5
*Zingiber officinale*	Zingiberaceae	Rhizomes	0.6
*Ziziphus spinachristi*	Rhamnaceae	Leaves	10.9

N.E: No Effect

**Table 2:** The IC50 values of the methanol extracts compared to eserine as a positive control.

Plants name	Family	IC50 value (mg/ml)
*Levisticum officinale*	Apiaceae	0.5
*Rheum ribes*	Polygonaceae	0.95
*Berberis integrimma*	Berberidacea	0.9
*Eserine*	*---*	0.8

### Kinetic analysis

**Fig. 1:**
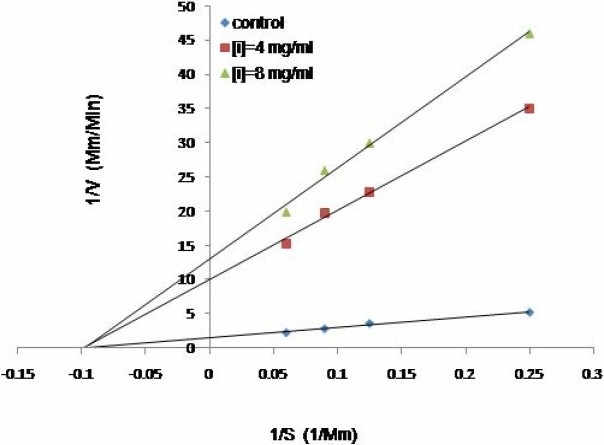
The Lineweaver-Burk plot of kinetic analysis of acetylcholinestrase at two different concentrations of *L. officinale* (4 and 8 mg/ml) in the presence of four different ATCI concentrations.

**Fig. 2:**
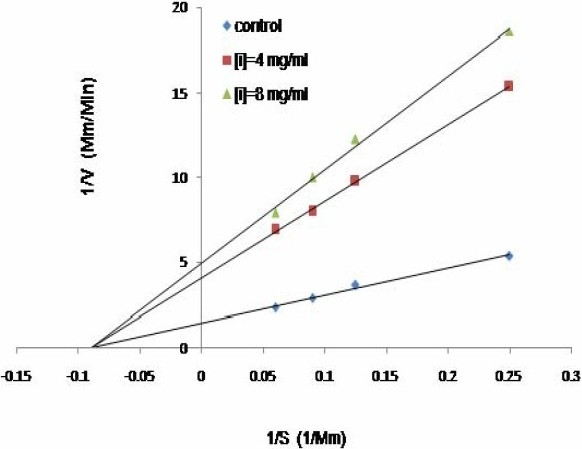
The Lineweaver-Burk plot of kinetic analysis of acetylcholinestrase at two different concentrations of *B. integrima* (4 and 8 mg/ml) in the presence of four different triolein concentrations.

**Fig. 3:**
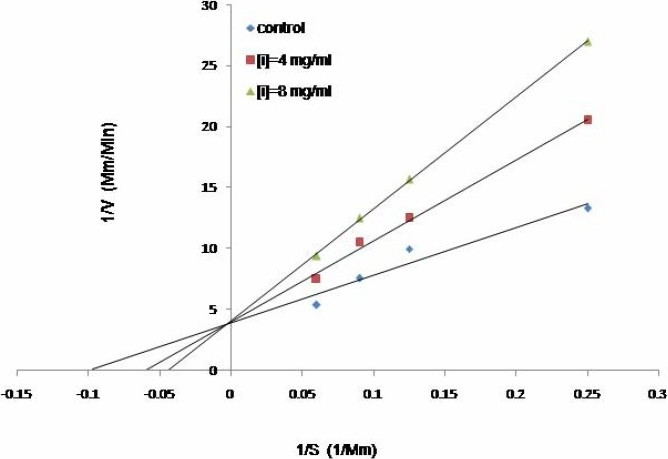
The Lineweaver-Burk plot of kinetic analysis of acetylcholinestrase at two different concentrations of *R. ribes* (4 and 8 mg/ml) in the presence of four different ATCI concentrations.

The inhibition modes of the three most active plant extracts were analyzed by doublereciprocal Lineweaver-Burk plot. *B. integrima* and *L. officinale* inhibited the enzyme activity in a non-competitive manner (Fig. 1 and 2), whereas *R. ribes* showed competitive inhibition (Fig. 3). The K_m_ value of the substrate, ATCI, for the Electric eel acetylcholinesterase was 9.4 mM and the V_max_ was 0.238 mM/min. When 8 mg/ml of each extract was added to the enzyme mixtures, the kinetics demonstrated competitive inhibition on enzyme activity by *R. ribes* with a V_max_ of 0.238 mM/min and a Km value of 21.2 mM. IC50 for *L. officinale, B. integrima* and *R.ribes* were 0.5, 0.9, and 0.95 mg/ml, respectively (Table 2). The Ki values of 1.6, 5.5 and 6.37 mg/ml were found for *L. officinale, B. integrima* and *R. ribes*, respectively.

## DISCUSSION

Acetylcholinesterase inhibitors are used for the treatment of Alzheimer’s disease. These inhibitors may interact with the central cholinergic system function to improve memory and cognitive disorders in the patients by decreasing the breakdown of acetylcholine in brain synapses (12). Nature is an unlimited resource for providing chemicals and biological compounds which are unique and complex insofar as their chemical synthesis seems impossible. The anti-cholinesterase activity of some plants in the world has been approved (12). In this study we concluded that roots of *L. officinale* and *B. integrima* and rhizomes of *R. ribes* possess a strong anticholinesterase activity. IC50 values (concentration required to inhibit 50% of enzyme activity) were calculated from the regression equation obtained from various concentrations of the test compounds (Table 2). Previously, it has been demonstrated (13) that the methanol extracts of *punica granatum* and *Rosa damascene* have more than 50% inhibitory effect on alpha manosidase activity, but these extracts did not show strong inhibitory effects on acetylcholinesterase. Extract of *L. officinale* exhibited strong inhibitory effects on the alpha glucosidase and the pancreatic lipase but exhibited weak inhibition on alpha manosidase (13,14). The plant extract demonstrated apoptotic activity on humans leukaemia cell line (15), and had an anti-mycobacterial activity as well (16). *B. integrima* produced no effect on the alpha glucosidase and the pancreatic lipase activity (14,17). 1-methylmalate, one of the *B. integrima* fruit components, increased the anti-microbial activity against *Staphylococcus aureus* (18). Isoquinoline alkaloids attained from the root of Turkish berberis species showed an anti-inflamatory and anti-nociceptive effects (19), whereas in our study this plant exhibited the anti-cholinesterase activity. *R. ribes* has shown to have hypo-glycemic effects in the alloxan induced diabetic rats but did not reveal hypoglycemic activity in healthy mice (20). It was also demonstrated that the herb possesses some anti-depressive activity (21). Some drugs such as rasagiline, used in the treatment of Alzheimer’s disease, retain the neuroprotective properties with their anti-cholinesterase and monoamine oxidase inhibitory effects and, has shown anti-depressant activity in animals (22). Therefore, the anti-depressant properties of *R. ribes* could be due to the anti-cholinesterase activity shown in this study. Other findings indicated that some components of *R. ribes* extract demonstrated selective cytotoxic activity on cancerous cell lines (23). The stem and root of *R. ribes* exhibited an antioxidant activity (24), but this plant did not have any inhibitory effect on the alpha glucosidase or the pancreatic lipase activity (14,17). The methanol extracts of *Rosa damascene, Quercus infectoria, Eucalyptus galbie, Myrtus communis, Terminalia chebulla, Punica granatum, Camellia sinensis*, and *Cinnamomum zeylanicum* showed more than 50 percent inhibitory activity on the pancreatic lipase or alpha glucosidase (14,17), while they revealed no or little effect on cholinesterase activity. One of the most important anti-cholinesterase drugs, tacrine, proved to have both competitive and non-competitive inhibitory activities on acetylcholinesterase (25). Tolserin, the novel experimental AD therapeutic agent, inhibits the acetylcholinesterase in a non-competitive manner (26). *R. ribes* showed some similarities in kinetic properties to tacrine. *L. officinale* and *B. integrima* were non-competitive inhibitors of the enzyme, as acting similar to tolserin. A competitive-inhibitor binds to the active site of the enzyme and affects K_m_ of the reaction. Components of *R. ribes* extract with competitive inhibition, may bind to the enzyme and block its activity. When an inhibitor binds to the enzyme and/or enzyme-substrate complex, it is considered as non-competitive inhibition where the inhibitor affects only the V_max_ of the reaction but has no effect on complex formation between the enzyme and the substrate. Therefore, two extracts that showed non-competitive inhibition on activity, probably have components that bind to enzyme or enzyme-substrate complex (27).

## CONCLUSION

According to our results, it is possible to assume that *R. ribes* may contain some components that are functionally or structurally similar to tacrine. The same might be true for *L. officinale* and *B. integrima* regarding the kinetic properties of the tolserin. Results of this study indicated that these plants may offer great potentials for the treatment of different diseases including AD, and their anti-acetylcholinesterase properties introduce them as promising candidates for more detailed *in vitro* and *in vivo* studies. Besides, these plants can be examined in order to isolate and identify the active ingredients, and this may serve as a foundation to find safer and more effective agent (s) for therapeutic use.
